# Biosynthesis of Gold Nanoparticles and Its Effect against *Pseudomonas aeruginosa*

**DOI:** 10.3390/molecules27248685

**Published:** 2022-12-08

**Authors:** Syed Ghazanfar Ali, Mohammad Jalal, Hilal Ahmad, Khalid Umar, Akil Ahmad, Mohammed B. Alshammari, Haris Manzoor Khan

**Affiliations:** 1 Department of Microbiology, Jawaharlal Nehru Medical College, Aligarh Muslim University, Aligarh 202002, India; 2 SRM Institute of Science and Technology, Kattankulathur, Chennai 603203, India; 3 School of Chemical Sciences, Universiti Sains Malaysia, Pulau Pinang 11800, Malaysia; 4 Department of Chemistry, College of Science and Humanities in Al-Kharj, Prince Sattam bin Abdulaziz University, Al-Kharj 11942, Saudi Arabia

**Keywords:** biofilm, GC-MS, gold nanoparticles, *Pseudomonas aeruginosa*, pyocyanin

## Abstract

Antimicrobial resistance has posed a serious health concern worldwide, which is mainly due to the excessive use of antibiotics. In this study, gold nanoparticles synthesized from the plant *Tinospora cordifolia* were used against multidrug-resistant *Pseudomonas aeruginosa*. The active components involved in the reduction and stabilization of gold nanoparticles were revealed by gas chromatography–mass spectrophotometry(GC-MS) of the stem extract of *Tinospora cordifolia.* Gold nanoparticles (TG-AuNPs) were effective against *P. aeruginosa* at different concentrations (50,100, and 150 µg/mL). TG-AuNPs effectively reduced the pyocyanin level by 63.1% in PAO1 and by 68.7% in clinical isolates at 150 µg/mL; similarly, swarming and swimming motilities decreased by 53.1% and 53.8% for PAO1 and 66.6% and 52.8% in clinical isolates, respectively. Biofilm production was also reduced, and at a maximum concentration of 150 µg/mL of TG-AuNPs a 59.09% reduction inPAO1 and 64.7% reduction in clinical isolates were observed. Lower concentrations of TG-AuNPs (100 and 50 µg/mL) also reduced the pyocyanin, biofilm, swarming, and swimming. Phenotypically, the downregulation of exopolysaccharide secretion from *P. aeruginosa* due to TG-AuNPs was observed on Congo red agar plates

## 1. Introduction

The emergence of antimicrobial resistance has become a serious health concern worldwide since the multidrug resistance in microorganisms has increased the morbidity and mortality rates worldwide [1,2,3,4]. The major problem with antibiotic therapy is that microorganisms develop resistance against antibiotics within a short span both in hospital- as well as in community-acquired infections. [5]. The resistance developed in microorganisms against the antibiotics poses a serious health challenge to treat infectious diseases, resulting in increased mortality [6]. Moreover, it is challenging to develop new antimicrobials or alternative therapeutics within a short span of time to treat pathogens [7,8,9]. *Pseudomonas aeruginosa* is one such kind of pathogen which develops resistance to antimicrobials and has been included in the list of ESKAPE pathogens, i.e., those pathogens which even surpass antibiotic treatment, therefore listed as critical priority pathogens [10,11]. A statistical survey report of 2019 from the United States Center for Disease Control and Prevention (CDC) states 32,600 cases and 2700 deaths from multidrug-resistant *P. aeruginosa* [12]

*P. aeruginosa* spreads its pathogenesis through different virulence factors such as pyocyanin, biofilm formation, and motility (pili and flagella).These virulence factors are responsible for attachment, colonization, and invasion into the host tissue, resulting in life threatening infection [13]. Pyocyanin cytotoxicity has already been reported, which involves pro-inflammation and free radical production, which cause cellular damage and necrosis [14,15,16]. The motility helps the microorganism to strive better in harsh environmental conditions, and it is an important virulence factor since it is necessary for proliferation, colonization, and infection [17]. Swarming and swimming in *P. aeruginosa* are different types of motilities [18]. Another virulence factor associated with *P. aeruginosa* is biofilm. A report from the United States National Institute of Health states that 80% of microbial infections are caused due to biofilm in the human body [19]. Biofilm can be formed on the respiratory system, reproductive organs, medical devices, etc. [20,21]. Exopolysaccharide (EPS) plays a crucial role in the development of biofilm; the EPS production allows irreversible attachment of *P. aeruginosa* on the surface, and it also allows social interaction, enhances gene transfer, and provides protection against antimicrobials [22]. Biofilm provides protection to microorganisms from harsh external environment, making them resistant. The main function of biofilm is to protect the microorganisms present within it from the harsh external environment and make them resistant [23].

Nanotechnology is an emerging field that is currently not only confined to physics or chemistry but has shown its promising applications in the field of medicine, specifically against microbial resistance. Nanomaterials are small-sized particles that have alarge surface area to volume ratio. Due to the large surface area to volume ratio, metal nanoparticles possess unique properties, some of which are of human interest, viz., treatment against bacterial infection [24]; some other biomedical applications include diagnostics, photothermal therapy, and electrical and optical sensing [25]. Gold nanoparticles are less toxic in nature and possess good compatibility with human cells in addition tobeing antimicrobial in nature [26]. Anticancer properties of gold nanoparticles have also been reported [27]. Enzymes such as acetylcholinesterase and butyrlcholinesterase when released in excess block the function of acetylcholine, which results in dementia. Some studies have claimed that gold nanoparticles downregulate the enzymes acetylcholinesterase and butyrlcholinesterase [28,29]. There are different methods of synthesis of nanoparticles, but the green method is preferred over chemical methods since chemical processes use harmful chemicals for reduction as well as for stabilization; moreover, the chemicals used pose a serious threat to the environment [30]. On the other hand, green synthesis, which uses green plants or parts of the plants, is an eco-friendly synthesis that does not use any chemicals [31]. The phytoconstituents from the plants act as reducing and stabilizing agents. Moreover, the use of plants does not pose any serious challenge, since their availability is abundant without any harmful effects.

*Tinospora cordifolia* (Willd.) Miers is a medicinal plant. The plant has been used traditionally for the treatment of fever, jaundice, chronic diarrhea, cancer, etc. [32].The stem of *T. cordifolia* has antidiabetic effects, since it regulates the blood glucose level in the body [33]. The extract from the roots of *T. cordifolia* possesses the ability to scavenge free radicals which are generated during aflatoxicosis [34].

Green synthesized nanoparticles (silver, zinc, etc.) from different plants possessing antibacterial, antivirulence, and antibiofilm potential have been well documented [35,36,37,38,39]. The synergistic effect of metal/metal oxide nanoparticles showing antibiosis has also been reported [40].

In view of the beneficial role of plants and medicinal properties of the stem of *Tinospora cordifolia*, we synthesized gold nanoparticles from the stem of *Tinospora cordifolia plant* [41] and further checked for antimicrobial activity and antivirulence against *P. aeruginosa.*

## 2. Result

The formation of gold nanoparticles from the stem extract of *Tinopora cordifolia* is represented by the equation below. The formation of gold nanoparticles in detailed view is shown as a flowchart diagram and attached as Supplementary Figure S1.

(1) $$\mathrm{Tinospora}\mathrm{Cordifolia}\;\mathrm{stem}\;\mathrm{Extract}+1\;\mathrm{mM}\;{\mathrm{AuCl}}_{3}\overset{\mathrm{After}\;24\;h}{\to }\mathrm{Gold}\;\mathrm{Nanoparticles}$$

### 2.1. SEM, TEM, and XRD Analyses

**Figure 1:**
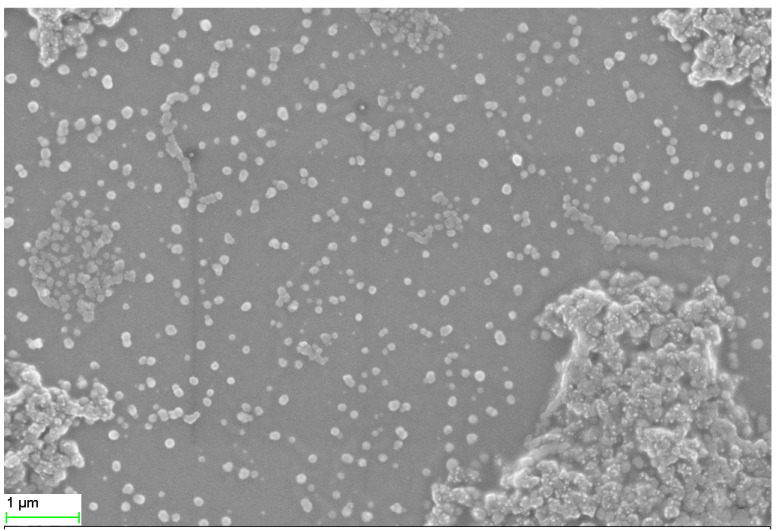
SEM image of TG-AuNPs.

**Figure 2:**
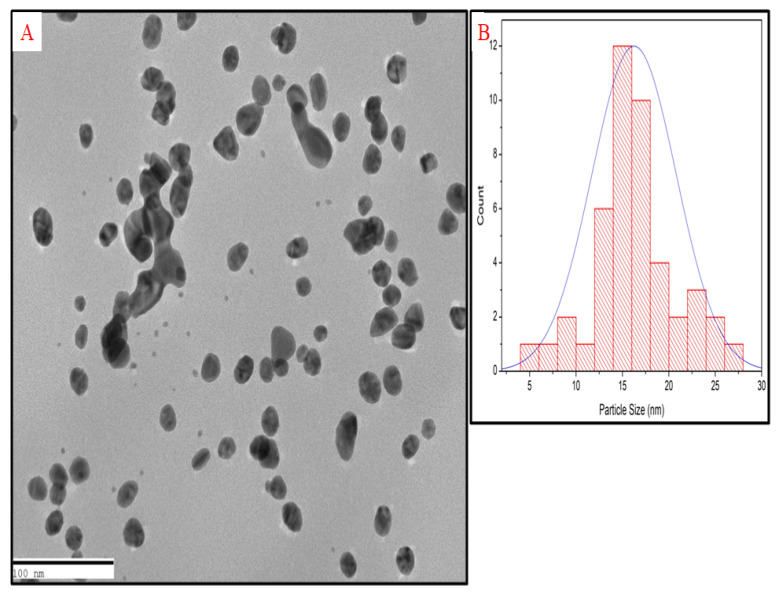
(**A**) TEM image of TG-AuNPs; (**B**) Particle size distribution of TG-AuNPs.

The TG-AuNPs as analyzed by SEM were poly dispersed and were of varying shape, but the majority of particles seemed to be spherical, whereas TEM analysis indicated the average particle size to be 16.25 nm(Figure 1 and Figure 2).

**Figure 3:**
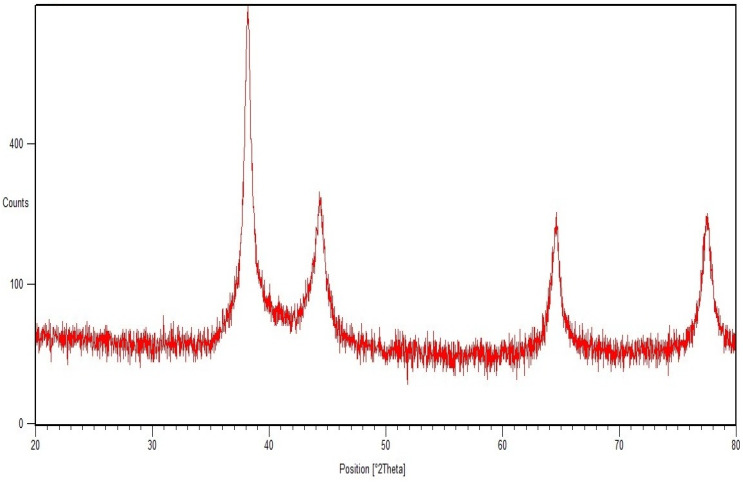
XRD of TG-AuNPs.

XRD analysis confirmed the crystalline nature of the gold nanoparticles. The respective diffraction peaks at 38.2°, 44.5°, 64.74°, and 77.6°, relating to (111), (200), (220), and (311) facets of the face-centered cubic (FCC) crystal lattice, correspond to pure gold (Figure 3) (JCPDS card no. 04-0784).

### 2.2. GC-MS of Tinospora Cordifolia Stem Extract

**Figure 4:**
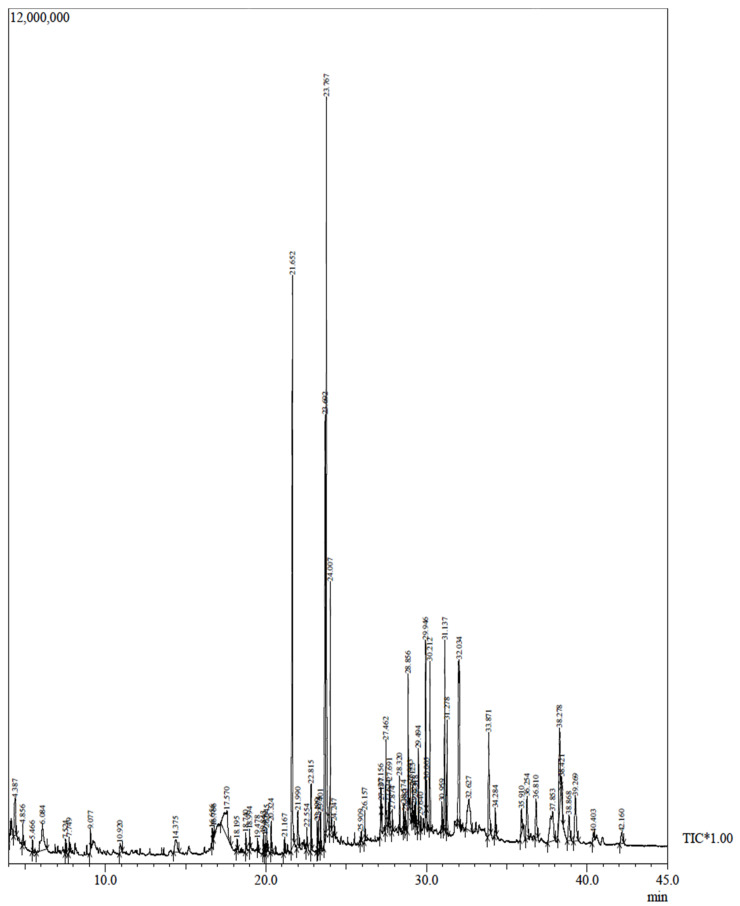
Representative GC-MS chromatogram of stem extract of *Tinospora cordifolia*.

The GC-MS of the methanolic stem extract of *Tinospora cordifolia* revealed 7-Tetradecanal (12.95%),n-Hexadecanoic acid (11.32%),9–12Octadecadienoic acid (10.39%), Benzene (5.97%), Pregna-5,16-dien-20-one,3-(acetyloxy)-16-methyle (3.85%), and Octadecanoic acid (3.40%) as the major components. The detailed analysis of GC-MS along with other compounds is shown in Table 1. The chromatogram reflecting different peaks obtained in the GC-MS analysis is shown in Figure 4.

### 2.3. Antibiotic Profile

*P*. *aeruginosa* (*n* = 10) were resistant to different antibiotics, and the details of antibiotics are the following: tobramycin (Tob, 10 µg,), piperacillin (Pi, 100 µg), nitrofurantoin (Nit, 300 µg), piperacillin-tazobactam (Pit, 100/10 µg), cefepime (Cpm, 30 µg),imipenem (Ipm, 10 µg), amikacin (Ak, 30 µg),ceftazidime (Caz, 30 µg),levofloxacin (Le, 5 µg), and sparfloxacin (Spx, 5 µg)

### 2.4. MIC of TG-AuNPs

The MIC of PAO1 was found to be 1000 µg/mL, whereas for all 10 clinical isolates the MICs varied:20% of the isolates showed an MIC of 1000 µg/mL, 50% of isolates showed an MIC of 1500 µg/mL; and 30% of isolates showed an MIC of 1800 µg/mL (Table 2). Three different concentrations, viz., 150,100, and 50 µg/mL, were considered for further antivirulence approaches.

#### 2.4.1. Effect of TG-AuNPs on Pyocyanin of *P. aeruginosa*

**Figure 5:**
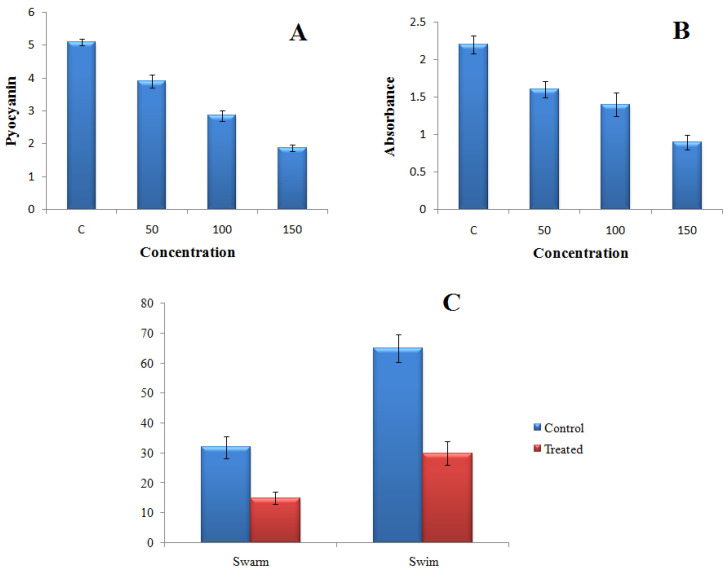
Representative of treated and untreated culture of PAO1 with TG-AuNPs: (**A**) pyocyanin; (**B**) biofilm; (**C**) motility (swarm and swim). For pyocyanin and biofilm 50, 100, and 150 µg/mL concentrations of TG-AuNPs were considered, whereas for motility only a 150 µg/mL of concentration of TG-AuNP was considered. Pyocyanin expressed as µg/mL. Absorbance measured at 595 nm. Swarm and Swim expressed as zone size in mm.

**Figure 6:**
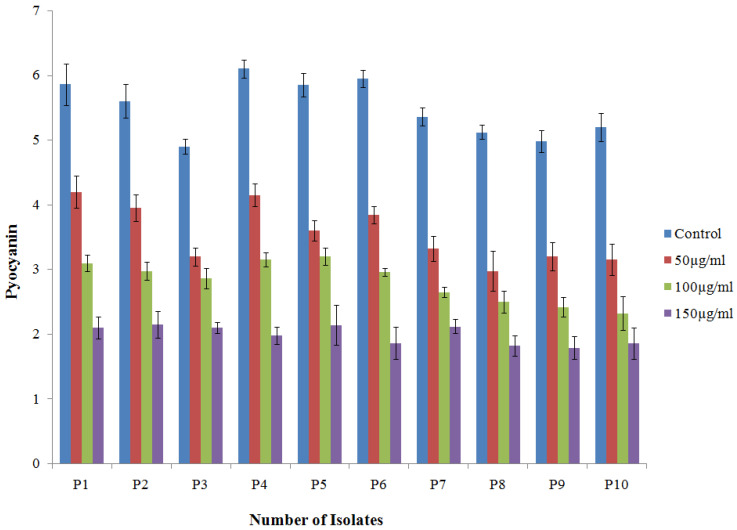
Bar graphs representative of level of pyocyanin after treatment of clinical isolates of *P. aeruginosa* with TG-AuNPs at 50, 100 and 150 µg/mL, along with control (untreated). Pyocyanin expressed as µg/mL.

Gold nanoparticles (TG-AuNPs) effectively downregulated the virulence of *P. aeruginosa*. In PAO1, a 63.1% reduction in the level of pyocyanin was observed at 150 µg/mL, whereas a similar concentration (150 µg/mL) of TG-AuNPs decreased the level of pyocyanin from 57.1% to 68.7% in clinical isolates. The lower concentration of 100 µg/mL caused a 43.9% reduction and 41.6% to 55.3% reduction in the level of pyocyanin for PAO1 and clinical isolates, respectively. The lowest concentration, i.e., 50 µg/mL of TG-AuNPs, caused 23.5% and 41.7% to 28.3% reductions in pyocyanin level for PAO1 and clinical isolates, respectively(Figure 5A and Figure 6).

#### 2.4.2. Effect of TG-AuNPs on Swarming and Swimming Motilities

**Figure 7:**
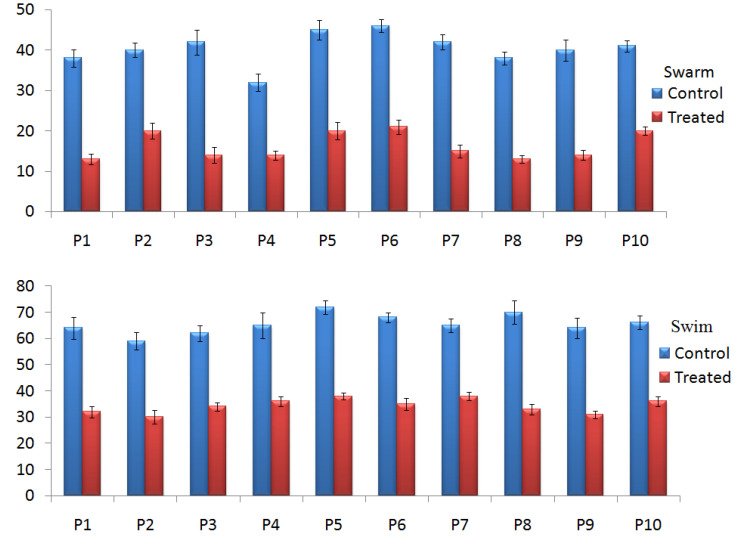
Bar graphs representative of swarm and swim after treatment of clinical isolates of *P. aeruginosa* with TG-AuNPsat150 µg/mL, along with control (untreated). Swarm and Swim expressed as zone size in mm.

**Figure 8:**
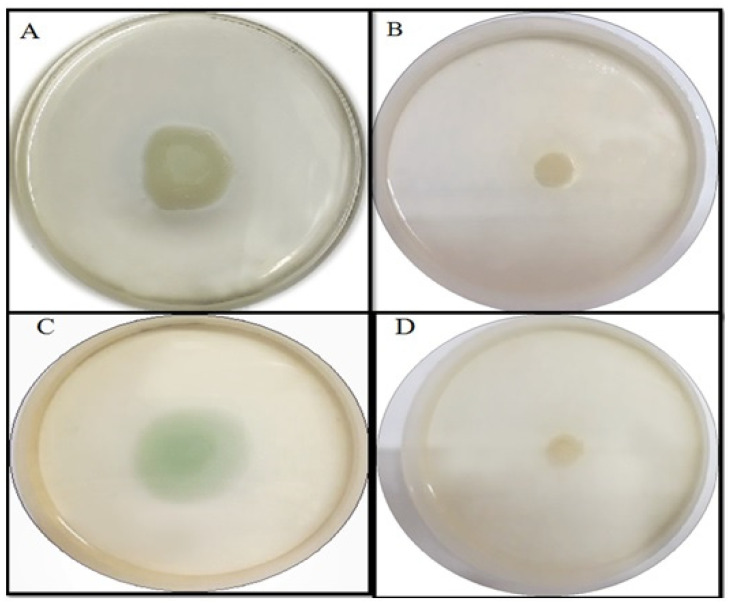
Representative of swarming of *P. aeruginosa*. (**A**) Swarm of PAO1. (**B**) Swarm of PAO1 after treatment with 150 µg/mL of TG-AuNPs. (**C**) Swarm of clinical isolate of *P. aeruginosa*. (**D**) Swarm of clinical isolate of *P. aeruginosa* after treatment with 150 µg/mL of TG-AuNPs.

**Figure 9:**
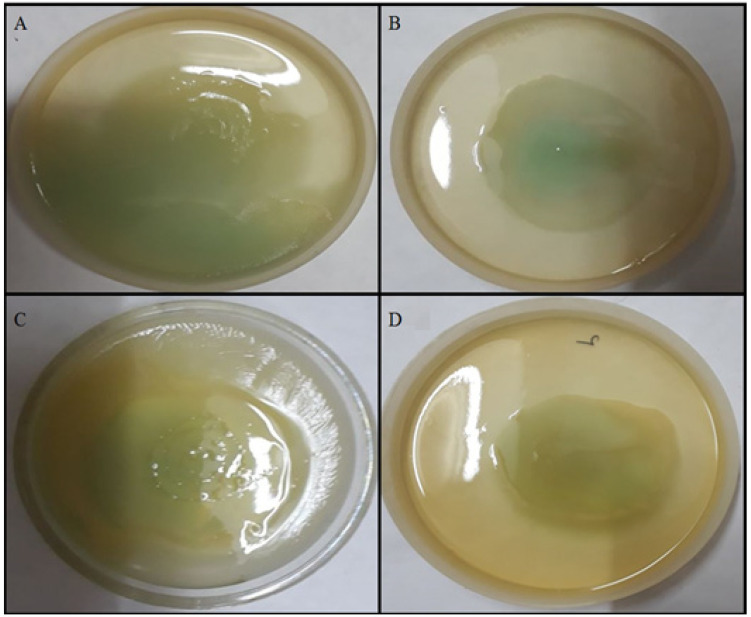
Representative of swimming of *P. aeruginosa*. (**A**) Swim of PAO1. (**B**) Swim of PAO1 after treatment with 150 µg/mL of TG-AuNPs. (**C**) Swim of clinical isolate of *P. aeruginosa*. (**D**) Swim of clinical isolate of *P. aeruginosa* after treatment with 150 µg/mL of TG-AuNPs.

The swarming and swimming motilities were also affected by the TG-AuNPs. Swarming and swimming motilities of PAO1 were reduced by 53.1% and 53.8% in the case of TG-AuNPs at 150 µg/mL (Figure 5C). Similar observations were also recorded for the clinical isolates. The reductions from 50% to 66.6% in swarming and 41.5 to 52.8% in swimming were observed at 150 µg/mL (Figure 7, Figure 8 and Figure 9).

#### 2.4.3. Effect of TG-AuNPs on the Biofilm by Crystal Violet Assay

**Figure 10:**
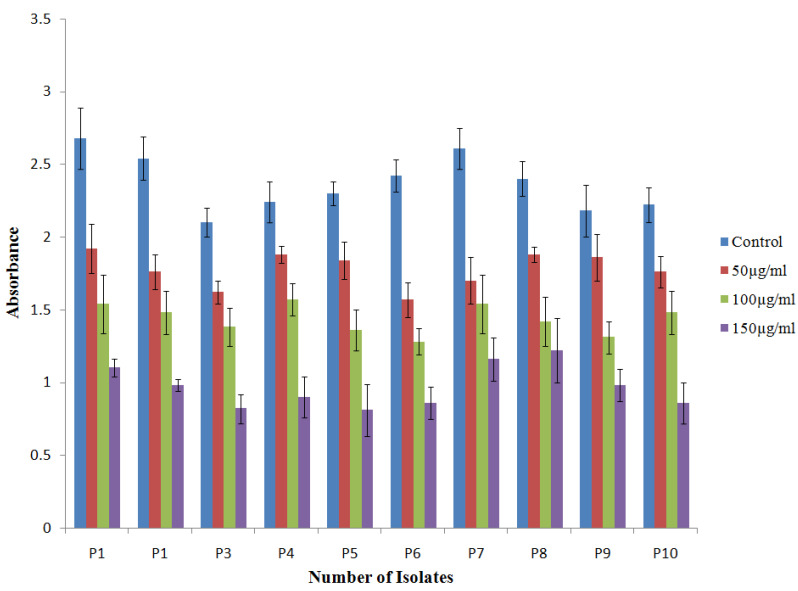
Bar graphs representative of biofilm after treatment of clinical isolates of *P. aeruginosa* with TG-AuNPs at 50,100, and 150 µg/mL, along with control (untreated). Absorbance measured at 595 nm.

Biofilm formation was also reduced at all three concentrations for PAO1, as well as for clinical isolates of *P. aeruginosa*. In PAO1, a 59.09% reduction in biofilm was observed at 150 µg/mL of TG-AuNPs, whereas a 49.1% to 64.7% reduction in biofilm formation was observed for clinical isolates of *P. aeruginosa* (Fig 5B). A lower concentration, i.e., 100 µg/mL, caused 36.3% and 29.9% to 47.1% reductions in biofilm formation forPAO1 and clinical isolates, respectively. Further, the lowest concentration, i.e., 50 µg/mL, effectively reduced the biofilm by 27.2% and 14.6% to 35.1% in PAO1 and clinical isolates, respectively (Figure 10).

#### 2.4.4. Effect of TG-AuNPs Using Congo Red Agar (CRA) Method

**Figure 11:**
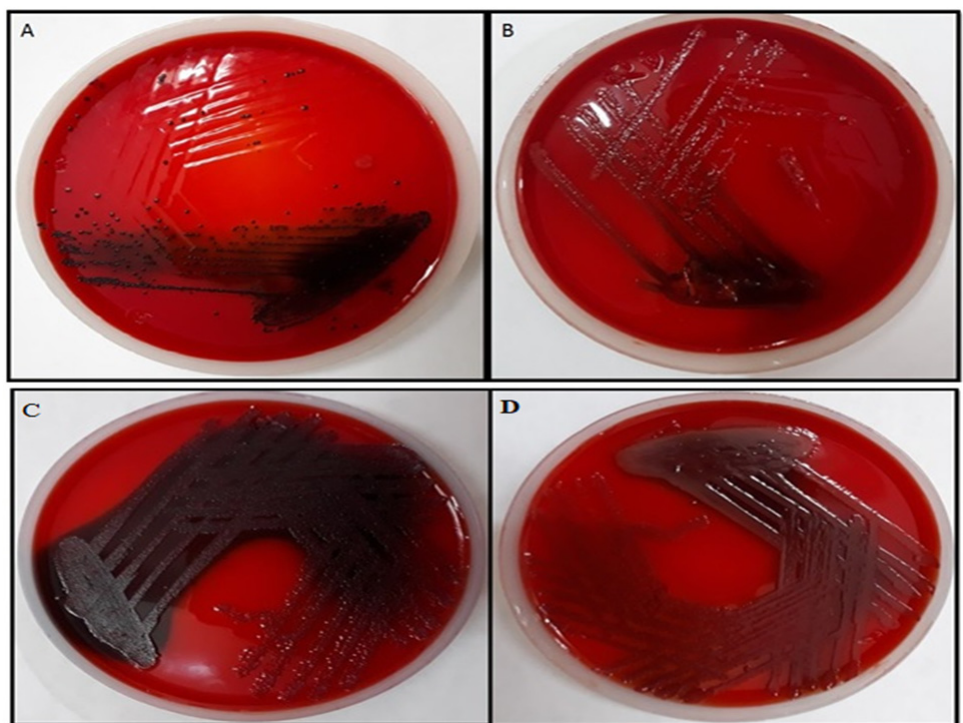
Representative of biofilm of *P. aeruginosa* on Congo red agar. Black coloration represents production of exopolysaccharide. (**A**) Biofilm of PAO1. (**B**) Biofilm of PAO1 after treatment with 150 µg/mL of TG-AuNPs. (**C**) Biofilm of clinical isolate of *P. aeruginosa*. (**D**) Biofilm of clinical isolate of *P. aeruginosa* after treatment with 150 µg/mL of TG-AuNPs.

TG-AuNPs at 150 µg/mL effectively reduced the exopolysaccharide production, which can be observed by the loss of black consistencies in colonies on Congo red agar plates amended with TG-AuNPs. The loss of black consistencies in PAO1 and clinical isolates of *P. aeruginosa* can be clearly seen when compared with the control (plates without TG-AuNPs) (Figure 11).

## 3. Discussion

The SEM analysis revealed that particles were polydispersed and not agglomerated. Since we can observe the surface morphology of nanoparticles through SEM, in order to better understand the size of nanoparticles TEM was performed, and it revealed the average particle size to be 16.25 nm. The histogram in Figure 2B represents the particle size distribution, which shows the varying size of nanoparticles. The methanolic stem extract of *Tinospora cordifolia* further revealed the presence of 7-Tetradecanal (12.95%), followed by n –Hexadecanoic acid (11.32%), 9,12-octadecadienoic acid (Z,Z) (10.39%), Benzene (5.97%), and Pregna-5,16-dien-20-one (3.85%). Some of the major components are shown in Table 1. We are of the opinion that 7 Tetradecanal and n–Hexadecanoic acid could be the major components responsible for the reduction inprecursor salt and stabilization of nanoparticles, although other components could also be responsible for the reduction and stabilization. Phytochemicals present in the plants reduce the metal ions, and the reduced metal ions are linked using atmospheric oxygen or from degrading phytochemicals. The phytochemicals also prevent the agglomeration of metal nanoparticles [42,43].

In our study, three different concentrations of TG-AuNPs (50, 100 and 150 µg/mL) were considered, which were lower than the MIC for PAO1 as well as for multidrug-resistant clinical isolates.

Pyocyanin, a major component involved in the pathogenesis of *P. aeruginosa*, allows the *P. aeruginosa* to coordinate and respond according to the change in environmental conditions [44]. In our study, the pyocyanin level was decreased for both PAO1 and multidrug-resistant clinical isolates. The maximum reductions of 63.1% for PAO1 and 57.1–68.7% for clinical isolates of *P. aeruginosa* for pyocyanin were observed at 150 µg/mL of TG-AuNPs. Lower concentrations, i.e., 100 and 50 µg/mL of TG-AuNPs, also caused reductions in the level of pyocyanin. Our results are in agreement with the previous studies, where 40–88% and 20–82% reductions were observed for the pyocyanin level at ½ and ¼ MIC of gold nanoparticles [45].

Swarming is a movement of bacteria (motility) that helps in colonization on the surface and helps in biofilm formation [46]. In addition to representing motility, the differentiation of swarm cells results in the alteration of metabolic bias and gene expression, indicating complex lifestyle adaptation [47,48]. When motility is regarding an aqueous solution, it is called swimming. The decrease in swarming and swimming motilities were also observed at 150 µg/mL. The decrease in the swarming and swimming motilities of *P. aeruginosa* both in PAO1 and clinical isolates are clearly observed in Figure 8 and Figure 9. In the plates without TG-AuNPs, more movement was observed in both swarm and swim, but in plates with TG-AuNPs restricted movement was seen. Swarming and swimming motility decreased by 53.1% and 53.8% for PAO1, whereas 50–66.6% and 41.5–52.8% reductions in swarming and swimming motility were observed for clinical isolates, respectively. At lower concentrations of 100 and 50 µg/mL of TG-AuNPs, zones of swarm and swim were not easy to measure, since they were equivalent to the control (untreated); therefore, we included only the 150 µg/mL concentration. Our results are supported by previous studies, where a complete reduction in swimming and approximately 30% and 50% reductions in swarming at 32 and 256 µg/mL of TG-AuNPs were observed [49]

One of the most important aspects of pathogenesis in *P. aeruginosa* is the formation of biofilm, through which the bacteria avoid the host immune response [50,51]. Biofilm is the aggregation of microbial communities and the site for the spread of infection. Further, the exopolysaccharide secretion forms the mask and does not allow the antimicrobial to penetrate [52].

Biofilm formation of PAO1 reduced by 59.09%, whereas a 49.1% to 64.7% reduction was observed for clinical isolates of *P aeruginosa* at 150 µg/mL. Lower concentrations of 100 and 50 µg/mL also caused a reduction in biofilm, both in PAO1 and clinical isolates. Our results are also in agreement with the previous studies of Elshaer and shaaban [45], where they have shown the downregulation of biofilm formation by 26–68% and 21–37% at ½ and ¼ MIC levels of gold nanoparticles. The loss of black consistency on the Congo red agar plate is the benchmark showing the decrease in EPS production. Our results showed the decrease in black consistency on Congo red agar plates amended with 150 µg/mL of TG-AuNPs both for PAO1 and for clinical isolates of *P. aeruginosa*, which is an indication of the loss of exopolysaccharide secretion (Figure 11). Our results are also in agreement with the previous studies, where baicalein fabricated nanoparticles reduced the exopolysaccharide secretion on Congo red agar plates [53]. Similar results showing the loss of exopolysaccharide production have been shown by Qais et al. [54].

## 4. Materials and Methods

All chemicals used are of ‘AR’ grade

### 4.1. Materials Used with Specification

- Stem of *Tinospora cordifolia*—for obtaining extract.
- Gold chloride (AuCl_3_), Sigma Aldrich (Germany)—salt for preparing gold nanoparticles.
- Methanol, Merck (Germany)—solvent used for extraction during GC-MS.
- Nutrient broth, Hi media (India)—liquid media for growth of bacteria.
- Nutrient agar, Hi media (India)—solid media for growth of bacteria.
- Chloroform, Merck (Germany)—used in pyocyanin extraction.
- Hydrochloric acid (HCl), Rankem (India)—used in pyocyanin extraction.
- Glucose, Rankem (India)—inoculated with nutrient media for swarming and swimming assay.
- Bacteriological agar, Hi media (India)—for solidifying liquid media.
- Crystal violet, Merck (Germany)—used in biofilm assay.
- Brain heart infusion, Hi media (India)—media used in Congo red biofilm assay.
- Sucrose, Rankem (India)—for analyzing biofilm using Congo red assay, since sucrose provides extra nutrients for growth of microorganisms.
- Congo red, Merck (Germany)—dye used in biofilm assay.

### 4.2. Synthesis of AuNPs

The gold nanoparticles were synthesized as previously described [41]. The part of the plant, i.e., stem, was collected from the nearby area of Aligarh, Uttar Pradesh, India. The stem consists of an outer husk, which was removed and sun-dried for few days until it became hard. The stem was then ground into powder form; the powder (10 gm) was then mixed with water (100 mL) and purified using filter paper. Furthermore, the centrifugation at 1200 rpm for 5 min allowed the removal of heavy biomaterials. The aqueous extract (10 mL) was mixed with 90 mL AuCl_3_ and left for 24 h.

### 4.3. Characterization of Nanoparticles

#### 4.3.1. Scanning Electron Microscopy (SEM)

The green synthesized gold nanoparticles (TG-AuNPs) were characterized using SEM (JSM 6510 LV) for analyzing morphology, as described by Ali et al. [41]. In brief, a drop of green synthesized gold nanoparticles (TG-AuNPs) was initially placed on the glass coverslip. The drop was allowed to dry on the glass coverslip at room temperature. After drying, the samples were placed under SEM and analyzed at an accelerating voltage of 15 kv and viewed on the screen attached to the SEM.

#### 4.3.2. Transmission Electron Microscopy (TEM)

TEM was used to analyze the size of TG-AuNPs, as previously described [41]. Briefly, a drop of gold nanoparticles (TG-AuNPs) was placed on a copper grid and left at room temperature for drying. After drying, the sample was placed in the TEM. Before viewing the vacuum was created, and the sample was illuminated with electronic radiations inside the TEM. The beam of the electron transmitted in the TEM allowed the detection of the sample on screen.

#### 4.3.3. X-ray Diffraction (XRD)

Gold nanoparticles were examined for crystalline or amorphous nature using XRD (Rigaku, Pittsburg, PA, USA) with a scanning 2 theta angle from 20° to 80° at 40 KeV.

### 4.4. GC-MS for Bioactive Compounds in Plant Extract

The GC-MS for bioactive compounds in plant extract was performed using a Shimadzu GC-MS-QP 2010 Plus fitted with an RTX-5 capillary column (60 m × 0.25 mm × 0.25 µm). Helium gas was used at 40.9 cm/s linear velocity. The oven temperature which was programmed at 90 °C was increased to 280 °C with a ramp rate of 10 °C/min. The total running time of GC was 50 min. The electron impact ionization method was applied with the ion source set at 230 °C. Methanol was the solvent used.

### 4.5. Bacterial Isolates

*P. aeruginosa* (*n* = 10) were isolated from the routine patient samples received in the Department of Microbiology J N Medical College & Hospital and were further identified using biochemical tests. The isolates were further tested for antibiotic sensitivity following the Clinical and Laboratory Standards institute guideline [55]. PAO1 was used as a control sample.

### 4.6. Determination of Minimum Inhibitory Concentration (MIC)

MIC was determined using the broth dilution method as previously described [56]. Briefly, overnight grown cultures of *P. aeruginosa* (PAO1 and clinical isolates) (2 × 10^6^ CFU/mL) were allowed to inoculate the nutrient broth with or without different concentrations of nanoparticles and were incubated at 37 °C for 24 h.

#### 4.6.1. Effect of TG-AuNPs on Pyocyanin

*P. aeruginosa* were inoculated with 5 mL nutrient broth in presence or absence of varying concentrations of TG-AuNPs at 150 rpm at 37 °C for 16 h in shaking incubator. Pyocyanin from *P. aeruginosa* treated or untreated with TG-AuNPs was extracted using 3 mL chloroform and then further re-extracted into 1 mL 0.2 NHCl until the color of the solution turned pink to deep red. Optical density at 520 nm multiplied by 17.070 determined the pyocyanin/mL of culture supernatant [57].

#### 4.6.2. Effect of TG-AuNPs on the Swarming Motility

Swarming of *P. aeruginosa* was analyzed by the procedure described by Chelvam et al. [58]. Semi-solid agar plates were prepared using nutrient broth and glucose (0.5%) mixed with bacteriological agar (0.5%). Before the pouring of media into plates, TG-AuNPs were added to the cooled media. Plates without TG-AuNPs were considered as control. After drying the plates, *P*. *aeruginosa* was spot inoculated on both the plates (with or without nanoparticles) and further incubated at 37 °C for 24 h.

#### 4.6.3. Effect of TG-AuNPs on Swimming Motility

Swimming was also checked by the procedure described by Chelvam et al. [58]. Semi-solid agar media constituting nutrient broth along with 0.25% bacteriological agar and 0.5% glucose were mixed, then autoclaved and cooled.TG-AuNPs were added before the pouring of media into the plates, and control plates were without TG-AuNPs. After drying, the spot inoculation of overnight grown *P. aeruginosa* was completed on the semi-solid agar plates including the plate without TG-AuNPs and incubated at 37 °C for 24 h.

### 4.7. Antibiofilm Potential of TG-AuNPs

#### 4.7.1. Effect of TG-AuNPs Using Crystal Violet Assay

Biofilm formation of *P. aeruginosa* by crystal violet assay was evaluated as previously described [59]. Briefly, 100µL (1 × 10^7^ CFU/mL) of mid-exponential *P. aeruginosa* culture was used to inoculate the tubes (2 mL) with or without TG-AuNPs. After inoculation, tubes were incubated at 70 rev/min for 24 h in shaking incubator. Tubes were then washed and stained with 0.1% *w*/*v* crystal violet for 30 min and then again washed three times, and finally filled with absolute ethanol and absorbance was recorded at 595 nm.

#### 4.7.2. Effect of TG-AuNPs Using Congo Red Assay

Antibiofilm efficacy of TG-AuNPs was observed by the method as described [38]. Briefly, brain heart infusion broth (37 g/L), sucrose (50 g/L), and bacteriological agar (10 g/L) were mixed and autoclaved, whereas Congo red agar solution (0.8 g/L) was autoclaved separately. After autoclaving and cooling, the Congo red agar solution was mixed with the brain heart infusion solution along with the desired concentration of TG-AuNPs and poured into the plates. Control plates were not amended with TG-AuNPs. *P. aeruginosa* was streaked on the control plates as well as on the plates amended with TG-AuNPs and incubated at 37 °C for 24 h.

## 5. Conclusions

In this paper, the green synthesized gold nanoparticles were used to target the virulence of multidrug-resistant *P aeruginosa*. The TG-AuNPs at very low concentrations (50,100, and 150µg/mL) were effective against the virulence factors of P. aeruginosa, viz., pyocyanin, swarming, swimming, and biofilm. The TG-AuNPs downregulated the pyocyanin production, along with the decrease in swarming and swimming motilities. The TG-AuNPs also lowered the biofilm formation, since it decreased the EPS production, which is a necessary requirement for biofilm. Finally, the GC-MS analysis of the plant extract showed the active component involved in the reduction and stabilization of TG-AuNPs. Finally, we are of the opinion that gold nanoparticles can be used as an alternative therapy at a very low concentration against multidrug-resistant microorganisms. Although the gold nanoparticles have shown their antivirulence effect at very low concentrations, extensive research on the toxicological aspect still needs to be conducted to better understand the effect of nanoparticles on different organs before they can be used inhuman applications.

## Figures and Tables

**Table 1:** Major components of GC-MS analysis of *Tinospora cordifolia* stem extract.

Peak	R. Time	Area	Area%	Name
1	23.767	27437724	12.95	7-Tetradecenal, (Z)-
2	21.652	23992314	11.32	n-Hexadecanoic acid, methyl ester
3	23.692	22020115	10.39	9,12-octadecadienoic acid (Z,Z)-
4	32.034	12640436	5.97	BENZENE, (2-ETHYL-4-METHYLE-1,3-PENTADIENYL)-
5	31.137	8164505	3.85	Pregna-5,16-dien-20-one, 3-(acetyloxy)-16-methyle-, (3.beta.)
6	24.007	7197068	3.40	Octadecanoic acid
7	33.871	6441213	3.04	Octacosanol
8	38.278	6350228	3.00	.gamma.-Sitosterol
9	37.853	5967832	2.82	1,4-METHANOAZULENE, DECAHYDRO-4,8,8-TRIMET
10	17.570	5954103	2.81	Inositol, 1-deoxy-

**Table 2:** MIC of PAO1 and clinical isolates of *P. aeruginosa*.

Standard (N = 1)		Clinical Isolate (N = 10)	
Isolate	MIC (μgmL^−1^)	Isolates	MIC (μgmL^−1^)
PAO1	1000	20%	1000
		50%	1500
		30%	1800

## Data Availability

Not applicable.

## Supplementary Materials

The following supporting information can be downloaded at: https://www.mdpi.com/article/10.3390/molecules27248685/s1, Figure S1: Flowchart for stepwise formation of gold nanoparticles.

## Sample Availability

Samples of the compounds are not available from the authors.
