# Supplementary material for: Biosynthesis of Gold Nanoparticles and Its Effect against Pseudomonas aeruginosa
Source: Molecules. 2022 Dec 8;27(24):8685. doi: 10.3390/molecules27248685 (PMC9781250; doi:10.3390/molecules27248685)
Supplement: Supplementary file 1 [file molecules-27-08685-s001.zip › molecules-2036130-supplementary.pdf]

Biosynthesis of Gold Nanoparticles and its Effect against *Pseudomonas aeruginosa*

Syed Ghazanfar Ali [1], Mohammad Jalal [1], Hilal Ahmad [2],Khalid Umar [3*], Akil Ahmad [4*], Mohammed B. Alshammari [4] and Haris M Khan [1]

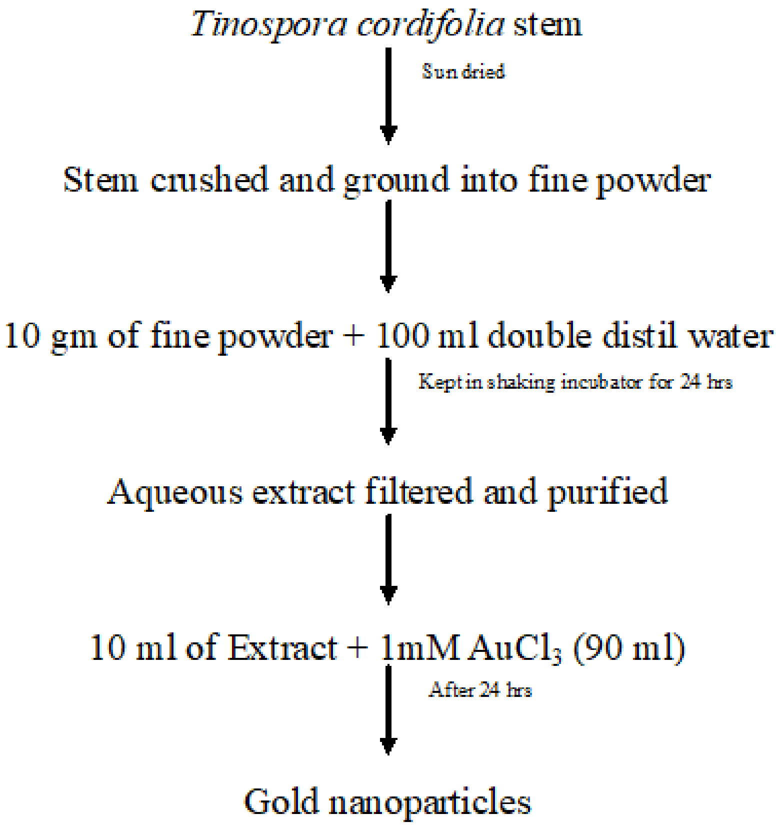

Figure S1. Flowchart for stepwise formation of gold nanoparticles
